# The correlation between phubbing and depression anxiety stress of first-year medical students: the mediating role of sedentary behavior

**DOI:** 10.3389/fpsyt.2026.1786645

**Published:** 2026-05-11

**Authors:** Mengyun Peng, Yingpeng Jiang, Pusen Lu, Na Li

**Affiliations:** 1 School of Nursing, Suzhou Medical College, Soochow University, Suzhou, China; 2 Suzhou Medical College, Soochow University, Suzhou, China; 3 Department of Nursing, The Second Hospital of Dalian Medical University, Dalian, China

**Keywords:** anxiety, depression, medical students, phubbing, sedentary, stress

## Abstract

**Background:**

Freshmen often experience challenges when adjusting from high school to college, which may elevate levels of depression, anxiety, and stress. This phenomenon is particularly pronounced in medical schools, where the overall academic atmosphere is widely regarded as exceptionally demanding and stressful.

**Objectives:**

To examine the indirect effect of sedentary behavior on the relationship between phubbing and depression anxiety stress of first-year medical students.

**Methods:**

This study conducted a cross-sectional survey with 795 first-year medical students from Soochow University in China from October 2024 to November 2024 by using electronic questionnaire. The instruments were Depression Anxiety Stress Scale-21 (DASS-21), Generic Scale of Phubbing (GSP), and Adolescent Sedentary Activity Questionnaire (ASAQ).

**Results:**

The findings indicate that first-year medical students’ phubbing and sedentary behavior positively affects their depression anxiety stress (r = 0.120 ~ 0.815, both *p* < 0.01), and phubbing positively impacts medical students’ sedentary behavior (r = 0.128, *p* < 0.01). Additionally, sedentary behavior acts as a significant mediator between phubbing and depression, anxiety, and stress. The indirect effect contributes to 1.9%~2.5% of the total effect.

**Conclusion:**

These findings indicate that reducing depression anxiety stress in first-year medical students can be achieved not only through direct improvements in phubbing but also through the indirect effects of reducing sedentary behavior.

## 1 Introduction

Transitioning from high school to college involves significant challenges, such as moving away from home, adjusting to a new environment, and improved independence. These changes can lead to first-year students’ feelings of loneliness, and homesickness, which may aggravate their emotional issues (1). Furthermore, due to the special nature of medicine, medical students are often faced with heavier and more complex knowledge and exams, since their depression anxiety and stress can be even higher (2, 3). A review of 21 articles investigating a total of 35,160 individual Chinese medical students found that the prevalence of depression ranged from 13.10 to 76.21% with a mean of 32.74%, and the prevalence of anxiety ranged from 8.54 to 88.30% with a mean of 27.22% (4). A study conducted across 22 medical schools in Brazil found that medical students had a significantly higher prevalence of depression and anxiety than non-medical students (5). Similarly, study from Pakistan reported high rates of mental health disorders among medical students (6). Common contributing factors included the pressure to pass exams and fear of entering the real world. Furthermore, a longitudinal study by Ludwig et al. on American medical students revealed that first-year medical students may particularly vulnerable to mental health issues (7). Students experiencing emotional distress require careful attention and management, as failure to cope effectively may lead to adverse consequences on both personal and professional levels (8, 9). Therefore, it is crucial to explore effective strategies to help first-year medical students adapt more smoothly and quickly to the new environment, thereby reducing the depression, anxiety, and stress levels.

Smartphones have become an indispensable part of daily life, functioning not only as tools for communication but also as platforms for information retrieval, entertainment, and business transactions. University students rely on smartphones to build social connections, access information, and engage in both academic and recreational activities (10). Consequently, given the widespread use of smartphones, excessive dependence on these devices has become increasingly prevalent among university students, leading to a phenomenon known as phubbing. Phubbing refers to the behavior of ignoring others by prioritizing one’s smartphone during conversations, thereby avoiding face-to-face interactions (11). This behavior should not be dismissed as trivial issue; rather, it represents a new and potentially harmful type of technological addiction that impacts both psychological and social well-being. For medical students, effective communication with patients and their families is a fundamental skill. However, phubbing may impede the development of these essential competencies (12). Additionally, excessive smartphone use has been reported to reduce individuals’ interest in other activities, leading to symptoms of anxiety, difficulty concentrating, and other negative effects on mental health when deprived of their devices (13, 14). Despite these concerns, the prevalence of phubbing among freshman medical students and its specific impact on depression, anxiety, and stress have yet to be fully established.

Sedentary behavior is defined as any waking behavior characterized by an energy expenditure ≤ 1.5 metabolic equivalents (METs), while in a sitting, reclining, or lying posture (15). Prolonged sedentary behavior has been related to an increased risk of physical health problems, including obesity and metabolic syndrome (16). Moreover, sedentary behavior has also been linked to a range of mental health disorders (17). Existing study suggests that sedentary behavior is prevalent among college students (18). A survey across eight Latin American countries found that sedentary behavior was common among medical students, with higher prevalence in the early years of study (19). Similarly, a study conducted among Japanese medical students reported that students’ sedentary time was greater than that of non-medical students (20). In a systematic review and meta-analysis, sedentary behaviour was categorized into three types: total sedentary behaviour, mentally active sedentary behaviour (e.g., reading, computer use, and social sedentary activities), and passive sedentary behaviour (e.g., watching television and listening to the radio) (21). The measurement methods in the included studies varied, including self-report questionnaires, accelerometer-based devices, and activity-specific self-reports (e.g., time spent on TV viewing, internet use, reading, and socializing). It is well established that sedentary behaviour is related to depression. Both phubbing and sedentary behavior reflect, to some extent, a reduction in social interactions and physical activity. However, the pathways through which these factors influence depression, anxiety, and stress among first-year medical students require further validation.

**Figure 1:**
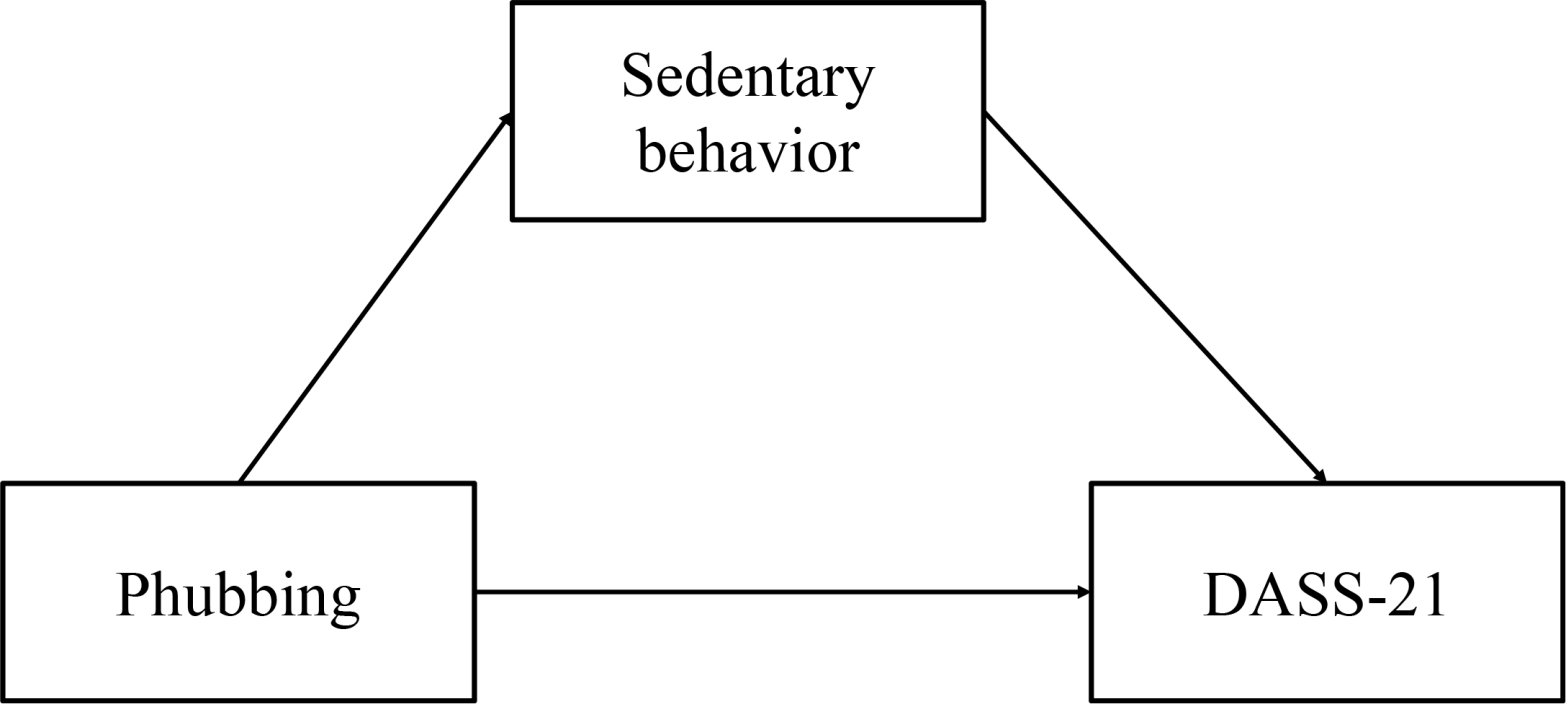
The theoretical model of this study.

Based on Engel’s Biopsychosocial Model (22), individual health is shaped by the interaction of biological, psychological, and social factors. Phubbing, which reflects a preference for smartphone use over face-to-face interaction, may undermine social connectedness and increase feelings of exclusion, contributing to psychological distress such as depression, anxiety, and stress. Sedentary behavior, often associated with excessive screen time, may further compound these effects by reducing physical activity and social engagement, both protective factors for mental health. Therefore, three hypotheses are proposed and hypothetical model presented in **Figure 1**.

H1: Phubbing is positively correlated with sedentary behavior.

H2: Sedentary behavior is positively correlated with depression anxiety stress.

H3: Sedentary behavior serves as a mediator in the relationship between phubbing and depression anxiety stress among first-year medical students.

## 2 Objectives

To explore the relationship between phubbing, sedentary behavior, and depression anxiety stress in first-year medical students, and examine the indirect effect of sedentary behavior.

## 3 Methods

### 3.1 Design and setting

A cross-sectional survey study.

### 3.2 Participants

This study employed convenience sampling. Participants were undergraduate medical students in their first year. There were no exclusion criteria. Larger sample sizes are recognized for producing more representative results. MacCallum et al. (23) recommend that the minimum sample size should be 100, or that the ratio of sample size to the number of independent variables should be at least 5. This study included a total of 37 independent variables; taking into account a 10% dropout rate, the minimum sample size was calculated to be 37 × 5 × 110% = 203. Given that a larger sample size is likely to provide a more representative sample of the population, this study ultimately distributed questionnaires to 1120 first-year medical students.

### 3.3 Measures

A questionnaire consisting of four sections, including demographic information, Depression Anxiety Stress Scale-21(DASS-21), Generic Scale of Phubbing (GSP), and Adolescent Sedentary Activity Questionnaire (ASAQ).

#### 3.3.1 Demographic information

Including gender, age, ethnicity, residence, smoking, drinking.

#### 3.3.2 Depression anxiety stress scale-21

The DASS-21 is a self-reported tool comprising 21 items, designed to assess three interconnected emotional states: depression, anxiety, and stress (24). Each subscale contains seven items, with participants rating each item on a scale from 0 to 3, where 0 indicates “not at all applicable to me” and 3 means “very much applicable to me.” The total score for each subscale is calculated by summing the responses and then multiplying by two. The classification for each emotional state is as follows: “mild” score is defined as 10–13 for depression, 8–9 for anxiety, and 15–18 for stress. “moderate” score is defined as 14–20 for depression, 10–14 for anxiety, and 19–25 for stress. “severe” score is defined as 21–27 for depression, 15–19 for anxiety, and 26–33 for stress. “extremely severe” score is defined as ≥28 for depression, ≥20 for anxiety, and ≥34 for stress. The Chinese version of DASS-21 demonstrates good validity and reliability (25). The Cronbach’s alpha coefficient for DASS-21 ranged from 0.863 to 0.912 in this study.

#### 3.3.3 Generic scale of phubbing

The Chinese version (26) of the GSP, initially developed by Chotpitayasunondh and Douglas (27), was modified to assess the degree to which individuals focus on smartphones while disregarding others in social contexts. This scale includes four factors: nomophobia, interpersonal conflict, self-isolation, and problem acknowledgment, with 15 items rated on a seven-point Likert scale. The Chinese version of GSP demonstrates good validity and reliability (26). The Cronbach’s alpha coefficient for GSP was 0.894 in this study.

#### 3.3.4 Adolescent sedentary activity questionnaire

The ASAQ was created by Hardy, Booth, and Okely (28), which aimed to evaluate sedentary behaviors. ASAQ includes 32 items covering various sedentary activities related to entertainment, education, travel, and social interactions. Respondents are asked to reflect on a typical week during the school term and report the amount of time they spend engaging in specific sedentary behaviors on weekdays and weekends. The total time spent on sedentary activities is calculated by summing the responses for weekdays and weekends, yielding an average daily sedentary time. A score of ≥ 4 hours per day is considered indicative of high sedentary behavior (28). The Chinese version of ASAQ demonstrates good validity and reliability (29). The Cronbach’s alpha coefficient for GSP was 0.885 in this study.

### 3.3 Data collection

This study was conducted online using “Wenjuanxing”. Our research team reached out to the dean of the medical school at Soochow University to explain the study’s purpose and process. The dean then forwarded the survey link to the class head-teacher, who distributed it to first-year medical students via WeChat. The first page of the questionnaire provided detailed information about the study and asked participants for consent to participate.

### 3.4 Data analysis

IBM SPSS version 19.0 was used. Descriptive statistics were applied to describe participant variables and characteristics. The mediating effect was tested through stepwise regression, and Pearson’s correlation coefficient was used to examine relationships between variables. In Step 1, phubbing (independent variable) was regressed on depression, anxiety, and stress (dependent variable). Step 2 involved regressing sedentary behavior on depression, anxiety, and stress. Step 3 regressed depression, anxiety, and stress on both phubbing and sedentary behavior. The link between phubbing and depression, anxiety, and stress became weaker (partial mediation) or non-significant (full mediation) when sedentary behavior was added, confirming a mediating effect. The indirect effect was estimated using a bootstrap approach with a 95% CI. Model 4 was implemented using the Process macro, and a p-value of less than 0.05 was considered statistically significant.

### 3.5 Ethical consideration

This study was conducted in accordance with the Declaration of Helsinki. The study was approved by Soochow University (number SUDA20241209H05).

## 4 Results

### 4.1 Common method bias test

The Harman one-way test identified 14 factors with eigenvalues exceeding one. The first factor accounted for 21.26% of the total variance, remaining below the suggested 40% threshold (30), indicating that common method bias is unlikely to affect the data analysis results.

### 4.2 Participants’ characteristics

A total of 1,120 participants were invited to complete the questionnaires, with 795 responses, resulting in a 71.0% response rate. As shown in **Table 1**, 434 of the 795 first-year medical students were male (54.6%). The students’ mean age was 18.25 years (SD = 0.71).

**Table 1:** Characteristics of the participants (*n*  = 795).

Variable	Category	Mean ± SD	Frequency	Percentage (%)
Gender	Female		361	45.4
	Male		434	54.6
Age		18.25 ± 0.71 (16-29)		
Ethnicity	Han		727	91.4
	Others		68	8.6
Residence	Urban		563	70.8
	Rural		232	29.2
Are you smoking?	Yes		13	1.6
	No		782	98.4
Are you drinking?	Yes		88	11.1
	No		707	88.9

### 4.3 Descriptive statistics for variables

First-year medical students showed moderate to high level of phubbing (mean = 45.55; SD = 13.33). The score of students’ sedentary behavior was (mean = 7.33; SD = 4.19), and 692 (87.0%) students showed high sedentary behavior levels. Stress is the most significant psychological issue among students’ depression, anxiety, and stress, with a score of (mean = 8.31; SD = 7.28) (**Table 2**).

**Table 2:** Descriptive statistics for variables (*n*  = 795).

Variable	Score	Frequency	Percentage (%)
Phubbing (Continuous)	45.55 ± 13.33		
Sedentary behavior (Continuous)	7.33 ± 4.19		
Sedentary behavior levels (Categorical)			
Low sedentary behavior levels (<4hrs)		103	13.0
High sedentary behavior levels (≥4hrs)		692	87.0
Depression (Continuous)	5.99 ± 6.90		
Depression Severity (Categorical)			
Normal		582	73.2
Mild		84	10.6
Moderate		99	12.5
Severe		17	2.1
Extremely severe		13	1.6
Anxiety (Continuous)	7.13 ± 6.58		
Anxiety Severity (Categorical)			
Normal		470	59.1
Mild		78	9.8
Moderate		146	18.4
Severe		63	7.9
Extremely severe		38	4.8
Stress (Continuous)	8.31 ± 7.28		
Stress Severity (Categorical)			
Normal		663	83.4
Mild		84	8.8
Moderate		99	4.5
Severe		17	2.1
Extremely severe		13	1.6

### 4.4 Correlations for variables

Depression, anxiety, and stress were positively correlated with phubbing (r = 0.399,0.424,0.449, all *p* < 0.001) and sedentary behavior (r = 0.128,0.120,0.144, all *p* < 0.01) (**Table 3**).

**Table 3:** Correlations for variables (*n*  = 795).

Variable	Phubbing	Sedentary behavior	Depression	Anxiety	Stress
Phubbing	1	–	–	–	–
Sedentary behavior	0.128^*^	1	–	–	–
Depression	0.399^*^	0.128^*^	1	–	–
Anxiety	0.424^*^	0.120^*^	0.770^*^	1	–
Stress	0.449^*^	0.144^*^	0.773^*^	0.815^*^	1

*p  < 0.01.

### 4.5 The indirect effect of sedentary behavior in phubbing and depression anxiety stress

The analysis revealed that both phubbing and sedentary behavior were closely associated with depression, anxiety, and stress. Subsequently, the study employed Process Macro Model 4 to examine the indirect effect of sedentary behavior. The findings indicated that phubbing was a significant positive predictor of depression, anxiety, and stress (c = 0.207,0.209,0.245, *p* < 0.01). Further analysis confirmed that even when sedentary behavior was taken into account, phubbing remained a significant predictor of depression, anxiety, and stress (c′ = 0.202,0.205,0.239, *p* < 0.01).

Additionally, phubbing positively predicted sedentary behavior (a = 0.040, *p* < 0.01), and sedentary behavior, in turn, was a significant positive predictor of depression, anxiety, and stress (b = 0.128,0.105,0.153, *p* < 0.01), which support Hypotheses 1 and 2.

**Figure 2:**
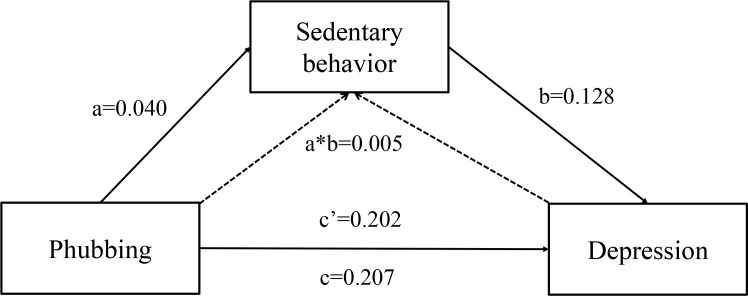
Mediating effect of sedentary behavior between phubbing and depression.

**Figure 3:**
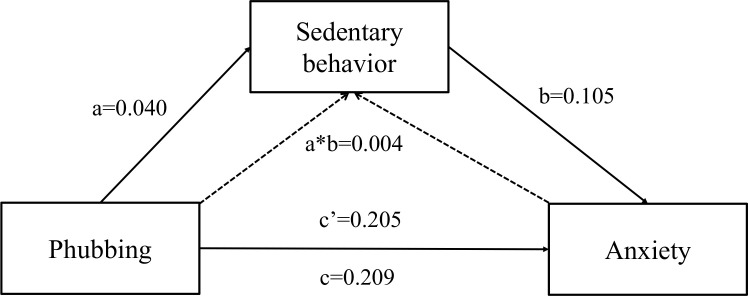
Mediating effect of sedentary behavior between phubbing and anxiety.

**Figure 4:**
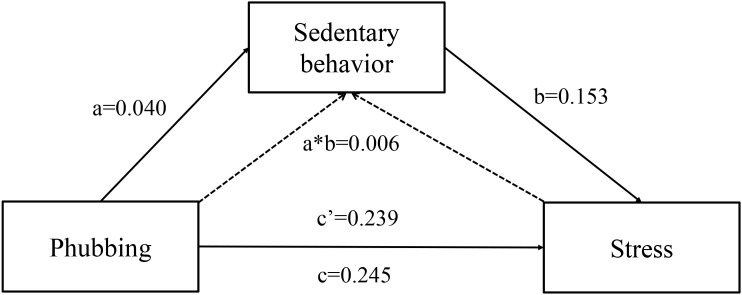
Mediating effect of sedentary behavior between phubbing and stress.

The percentile bootstrap method with bias correction further validated that phubbing played a significant indirect effect. The indirect effect was 0.006, 0.004,0.006, with a 95% CI of (0.001, 0.011; 0.001, 0.009; 0.002, 0.012), accounting for 2.4%, 1.9%, 2.5% of the total effect separately, thereby supporting Hypothesis 3. **Figures 2**–**4** and **Tables 4**–**6** present the direct, indirect, and total effects of sedentary behavior.

**Table 4:** Bootstrap mediation effect test results for phubbing, sedentary behavior, and depression.

Relation of efficacy	Efficiency value	SE	LLCI	ULCI	Percentage of efficiency
Total effect	0.207	0.017	0.174	0.239	–
Direct effect	0.202	0.017	0.168	0.234	97.6%
Indirect effect	0.006	0.003	0.001	0.011	2.4%

**Table 5:** Bootstrap mediation effect test results for phubbing, sedentary behavior, and anxiety.

Relation of efficacy	Efficiency value	SE	LLCI	ULCI	Percentage of efficiency
Total effect	0.209	0.016	0.178	0.240	–
Direct effect	0.205	0.016	0.174	0.236	98.1%
Indirect effect	0.004	0.002	0.001	0.009	1.9%

**Table 6:** Bootstrap mediation effect test results for phubbing, sedentary behavior, and stress.

Relation of efficacy	Efficiency value	SE	LLCI	ULCI	Percentage of efficiency
Total effect	0.245	0.017	0.211	0.279	–
Direct effect	0.239	0.017	0.205	0.273	97.5%
Indirect effect	0.006	0.003	0.002	0.012	2.5%

The results of the three models, after controlling for age and gender, are shown in **Tables 7**, **8**, and **9**. In Model 1, the analysis revealed that phubbing had a positive impact on sedentary (β = 0.405, t = 3.628, *p* < 0.01). In Model 2, a positive correlation was observed between sedentary behavior and depression, anxiety, and stress (β = 0.208, 0.188, 0.248, t = 3.592,3.391,4.056, *p* < 0.01). In Model 3, the interaction between phubbing and sedentary behavior demonstrated a significant positive effect on depression, anxiety, and stress (β = 0.103~0.241, t = 2.039~13.798, *p* < 0.01). Moreover, sedentary behavior served as a partial mediator in this relationship.

**Table 7:** Modelling of intermediation effects for phubbing, sedentary behavior, and depression.

Predicative variable	Model 1		Model 2		Model 3	
	β	t	β	t	β	t
Gender	2.701	2.871	-0.831	-1.701	-1.389	-3.080
Age	0.584	0.880	0.316	0.916	0.195	0.616
Phubbing→Sedentary behavior	0.405	3.628				
Sedentary behavior→Depression			0.208	3.592		
Phubbing + Sedentary behavior→Depression					0.207/0.124	12.180/2.322
R^2^	0.027		0.021		0.176	
F	7.358*		5.717*		42.176*	

*p  < 0.01, All coefficients are standardized (β).

**Table 8:** Modelling of intermediation effects for phubbing, sedentary behavior, and anxiety.

Predicative variable	Model 1		Model 2		Model 3	
	β	t	β	t	β	t
Gender	2.701	2.871	-0.381	-0.815	-0.944	-2.214
Age	0.584	0.880	0.189	0.575	0.068	0.226
Phubbing→Sedentary behavior	0.405	3.628				
Sedentary behavior→Anxiety			0.188	3.391		
Phubbing + Sedentary behavior→Anxiety					0.208/0.103	13.004/2.039
R^2^	0.027		0.016		0.189	
F	7.358*		4.242*		46.133*	

*p  < 0.01, All coefficients are standardized (β).

**Table 9:** Modelling of intermediation effects for phubbing, sedentary behavior, and stress.

Predicative variable	Model 1		Model 2		Model 3	
	β	t	β	t	β	t
Gender	2.701	2.871	-0.015	-0.029	-0.667	-1.434
Age	0.584	0.880	0.372	1.025	0.231	0.708
Phubbing→Sedentary behavior	0.405	3.628				
Sedentary behavior→Stress			0.248	4.056		
Phubbing+ Sedentary behavior→Stress					0.241/0.150	13.798/2.713
R^2^	0.027		0.022		0.212	
F	7.358*		5.942*		53.119*	

*p  < 0.01, All coefficients are standardized (β).

## 5 Discussion

This study proposes alleviating first-year medical students’ depression anxiety stress from phubbing and sedentary behavior. As we know, this is the first study to examine the sedentary behavior in the association of phubbing and depression anxiety stress in first-year medical students. The rate of depression anxiety stress that occurs among students is not low. Phubbing and sedentary behavior are positively associated depression anxiety stress in freshmen, and sedentary behavior partially mediates this relationship.

Medical students frequently encounter more challenges due to the unique demands of their profession (2, 3). This study showed that the prevalence rates of depression, anxiety, stress among first-year medical students were 26.7%, 40.95%, and 16.6%, respectively, which are comparable to the depression rates reported in the Peru (31), but significantly lower than those observed in Pakistan (6). A potential explanation for this discrepancy lies in the differences between the study populations. The participants in the Pakistani study were predominantly from private schools, while those in this study were from public. The differences in examination standards and academic pressures between private and public schools may account for this variation. Medical students in private schools often face greater career-related stress and higher family expectations regarding academic performance. The stress levels among first-year medical students were the lowest in this study, which may be explained by the timing of the study. Conducted in October, just one month after the start of the academic year, most freshmen had only a limited exposure to specialized and core courses. The relatively lighter course schedule during this early stage likely contributed to lower academic stress compared to their levels of depression and anxiety.

Phubbing refers to the behavior of ignoring others by prioritizing one’s smartphone during conversations, thereby avoiding face-to-face interaction (11). Phubbing is not only a result of technological advances, but also a whole new aspect of social behavior that impacts student achievement and developmental sustainability (32). Previous studies have shown that phubbing is strongly linked to behavioral, emotional, and academic performance among undergraduate and graduate students (33). Numerous studies in educational contexts show a high prevalence of phubbing and its negative impact on students (34). This study showed moderate to high level of phubbing in first-year medical students and positively correlated with depression anxiety stress, which is consistent with previous study (35, 36). Phubbing often leads to reduced face-to-face interactions, weakening social connections and increasing loneliness, which are strongly linked to depression and anxiety. Freshmen may overuse their phones to escape academic pressure or negative emotions, creating a vicious cycle where reliance on devices exacerbates mental health issues. To manage phubbing behavior, we recommend organizing workshops for first-year medical students to raise awareness of phubbing and its potential negative impact on academic and professional success.

First-year students, having just entered university, may not yet have fully adapted to campus life or developed effective time management skills. As a result, they are likely to spend more time engaged in indoor activities such as studying or using electronic devices, which may increase their risk of sedentary behavior (37). This study found a high prevalence of sedentary behavior (87.0%) among first-year medical students, with a positive correlation between sedentary behavior and depression, anxiety, and stress, aligning with findings from the Finland study (38). From a biological perspective, prolonged sedentary behavior may disrupt neurotransmitters like serotonin and dopamine, which are crucial for mood regulation (39). Conversely, from a sociological perspective, sedentary behavior may reduce face-to-face interactions, foster feelings of loneliness and increase the risk of mental health (40). Addressing first-year medical students’ sedentary behavior through regular physical activity, social engagement, and time management strategies may help mitigate its adverse effects on mental health.

Another key finding from this study is that sedentary behavior mediates the relationship between phubbing and depression, anxiety, and stress. This suggests that phubbing not only has a direct impact on these negative emotional states but also exerts an indirect influence through increased sedentary behavior. Both phubbing and sedentary behavior reflect, to some extent, a reduction in social interaction and physical activity, pointing to a growing reliance on information technology. While the use of smartphones or computers may serve as a short-term coping mechanism to alleviate stress or negative emotions, excessive and prolonged use can lead to problematic behaviors, including behavioral addiction (41).

However, it is important to note that the mediating effect of sedentary behavior observed in this study was relatively small. Specifically, the indirect effect via phubbing accounted for only 2% of the total effect. This indicates that while sedentary behavior plays a role, it does not fully explain the psychological impact of phubbing. The practical significance of this finding is negligible, and the small effect size suggests that reducing sedentary behavior alone may not meaningfully alleviate phubbing-related mental health issues. The modest size of the mediation effect suggests that disrupted interpersonal relationships, poor sleep quality, or reduced self-esteem, which may also contribute to the development of depression, anxiety, and stress. For first-year medical students, who often face high academic pressure and may lack sufficient self-regulation and time management skills, even a small mediating effect can have cumulative consequences over time.

Therefore, intervention strategies should not only aim to reduce phubbing and sedentary behavior but also address broader psychosocial factors. Promoting balanced digital habits, enhancing social connectedness, and fostering emotional regulation skills may provide a more comprehensive approach to improving mental health among first-year medical students.

### 5.1 Limitation

This study has several limitations. First, this study has several limitations. First, the cross-sectional study design does not allow for inferring causal relationships or directionality between phubbing, sedentary behavior and depression, anxiety, and stress. Although we proposed a conceptual model based on a theoretical framework, the observed indirect effects were small; therefore, the results should be regarded as exploratory explanatory pathways. Future studies should adopt longitudinal or experimental designs to test the proposed directional relationships and formally establish the temporal sequence. Second, data collected through self-reported online surveys may be subject to recall bias. Third, participants were from a single university and using convince sampling methods, which may limit generalizability and introduce selection bias. Future research could expand the sample and examine differences across cultural and educational contexts. Furth, the ASAQ measures total sedentary time but does not differentiate between screen-based (e.g., smartphone use) and non-screen-based (e.g., studying, reading) activities. This is a limitation because the proposed mediation pathway specifically involves phubbing (smartphone-related behavior), and the indirect effect might be stronger if screen-based sedentary behavior were measured separately. We therefore recommend that future studies use domain-specific sedentary behavior measures to distinguish screen-based from non-screen-based sedentary time. Fifth, this study controlled only for age and gender, other potential confounders were not measured, such as total screen time. These unmeasured factors may confound the observed associations. Future studies should include more covariates to better isolate the specific role of phubbing and sedentary behavior in psychological distress.

## 6 Conclusion

This study revealed a significant association between phubbing, sedentary behavior, and depression anxiety stress of first-year medical students. Sedentary behavior was found to mediate this relationship, emphasizing the need for support for first-year medical students who exhibit tendencies toward phubbing and sedentary behaviors, as such support is critical for alleviating levels of depression, anxiety, and stress during the high school-to-college transition. Furthermore, the study suggests that schools should implement comprehensive student development programs, which could include increasing outdoor activities to reduce the frequency of smartphone or computer use, fostering social interactions among students, and mitigating negative emotions. These insights are valuable for foundational research aimed at helping first-year medical students adapt quickly to new environments and learning modes, as well as for guiding future efforts to create a positive learning atmosphere for incoming students.

## Data Availability

The original contributions presented in the study are included in the article/supplementary material. Further inquiries can be directed to the corresponding author.
